# Illicit drug use in university students in the UK and Ireland: a PRISMA-guided scoping review

**DOI:** 10.1186/s13011-023-00526-1

**Published:** 2023-03-14

**Authors:** Maeve Boden, Ed Day

**Affiliations:** University of Birmingham, Institute for Mental Health, School of Psychology, 52 Pritchatts Road, Edgbaston, Birmingham, B152TT UK

**Keywords:** Psychoactive drugs, Students, University, Scoping review

## Abstract

**Background:**

Interest in the health and well-being of university students has increased in the UK and Ireland in the past two decades as their numbers have grown. Recent high-profile deaths of students after using illicit drugs have highlighted the importance of the topic for policy makers. This scoping review maps the state of the existing literature evaluating use of illicit drugs in university students in the UK and Ireland. It aims to highlight research gaps and inform policy.

**Method:**

We conducted a systematic search of papers related to psychoactive drug use in university students in the UK and Ireland published before August 2021. The 18 extracted study characteristics included author(s); year of publication; journal; location of data collection; study design; delivery method (e.g., online survey, in-person, postal survey); number of participants; response rate; participant course of study, year of study, degree level (i.e., undergraduate, postgraduate), gender and age; time-period assessed (e.g., lifetime, current use, past 12 months); primary aim; primary outcome; ethical approval; and funding source.

**Results:**

The PRISMA-guided search strategy identified 1583 papers for abstract review; of 110 papers retained for full-text review, 54 studies met criteria for inclusion for this paper. Primary outcomes were coded into five groups: prevalence and patterns of drug use; factors associated with drug use; attitudes and knowledge about, and motivation for, drug use; supply of drugs; consequences of drug use. The results show that there is no coherent body of research in this area. The prevalence of reported drug use has crept up and the range of substances reported has broadened over time, and attitudes to drugs on average have normalised. However, there are significant methodological limitations that limit the utility of these findings. There was little evidence of published work on prevention of, or intervention to reduce, drug-related harms.

**Conclusion:**

The domains identified offer a framework for university administrators, researchers and policy makers to understand the potential response to drug use in university students in the UK and Ireland. Recommendations are made to fill the gaps in the research evidence base.

**Supplementary Information:**

The online version contains supplementary material available at 10.1186/s13011-023-00526-1.

## Background

Illicit drugs are psychoactive substances whose non-medical use has been banned by international drug control treaties as they are believed to pose an unacceptable risk to the health of people that use them [1]. Prospective cohort studies in high-income countries consistently show that adolescence is the peak period for first illicit drug use, and levels and frequency of use begin to increase in mid-adolescence and peak in early adulthood before slowly declining with age [2, 3]. This is consistent with the latest figures available for England and Wales, which relate to trends in drug use for the year ending March 2020 [4]. Approximately 1 in 5 (21%) young adults aged 16–24 years had taken an illicit drug in the last year (1.3 million people) compared with 1 in 11 (9.4%) adults aged 16–59 years. Furthermore, twice as many young adults had taken a drug more than once a month in the last year. Cannabis was the most common drug used by 16–24 year olds (18.7%).

Illicit drug use in young adults tends to be more experimental and opportunistic than in older age groups, but some young adults start to use drugs more frequently and a small number progress to regular use and dependence. Degenhardt and colleagues have described the epidemiology of illicit drug use in young people (defined as 10–24 years old) around the world, the harms that they cause, and the potential responses available to reduce these harms [2, 5, 6]. Variations in patterns of drug use initiation between countries and cultures suggests that a young person’s entry into illicit drug use may reflect their personal characteristics, illicit drug availability, and social settings that facilitate or deter drug use [2].

Illicit drug use appears to be a common but infrequent activity amongst university students. In the UK most students start university at the age of 18 or 19, and it was reported in 2017/18 that a record 50.2% of English 17- to 30-year-olds had participated in higher education. This coincides with a period often known as ‘emerging adulthood’, commonly defined as the period between the end of compulsory schooling and the onset of adult commitments such as employment, long-term sexual relationships and parenthood [7].

During this period most students live away from home for the first time and so become more financially independent and self-reliant as a consequence. New friends are made and old friends from school are left behind, as the individual begins to forge a new adult identity away from parental influence. Peer and romantic interactions become more important, and there is a need to be more self-directed in terms of time management. The university can therefore be seen as a specific ‘risk environment’ [8], where cultural and environmental factors including distance from parents and the interconnected nature of student life can accelerate trajectories from drug experimentation to more involved drug use [9]. In this transitional phase, experimentation with drugs may be seen as a normative behaviour by students that helps them to develop new social relationships, enhance new experiences or to boost academic or recreational performance [7, 10].

A national survey of 2810 students in the UK in 2018 reported that 56% of respondents had used drugs, and 39% currently used them [11]. Cannabis was the most frequently taken drug (94% of respondents who said that they had used drugs) and was the most likely to have been used regularly. However, ecstasy, nitrous oxide and cocaine had all been used by most of the drug-using population at some point. Large scale North American surveys show that the annual prevalence of illicit substance use in university student populations has grown gradually from 34% in 2006, to 43% in 2018 [12]. The US national Monitoring the Future follow-up study reported that the annual prevalence in cannabis use in university students was at a historic high level, with a 5-year trend from 2014–2019 showing a significant 8.6% increase [12].

The 2017 Government Drug Strategy in England emphasised that Colleges and Universities had an important role to play in supporting the health and welfare of their students [13] (p9). Likewise, when the Irish Government convened a Rapid Response Group in September 2019 to address illicit drug use in higher education institutions (HEIs) it noted that HEIs “*can assist in addressing the hazards of illicit drug use by implementing actions that have the potential to reduce the number of students who decide to use drugs in the first place, or to reduce the harm experienced by those students who have chosen to use drugs*” [14]. However, there has been relatively little research on the incidence and prevalence of drug use in UK or Irish university student populations. Previous reviewers have noted methodological shortcomings, including small sample sizes and/or a narrow focus on students from a single university or even a single department [15, 16]. It is not clear whether student attitudes to drugs differ from their non-student peers, or whether they have changed over time. There is also a lack of consensus on the extent of drug-related harms and the most effective strategies to reduce them if necessary. In early 2022 Universities UK (UUK) announced that it wished to set out a common approach to reduce harms from drug use and to better tackle supply [17]. They noted that some universities had the stated aim of a ‘drug-free campus’ whereas others had implemented harm reduction and treatment services.

A coherent body of research into illicit drug use by university students might be expected to explore the epidemiology of use, potential mechanisms of initiation, escalation and reduction in use, prevailing attitudes towards and beliefs about drugs, any potential benefits or harms resulting from use, and methods for detecting, preventing and treating problematic use. The existing evidence base in this area is built on research in North American university populations [18, 19], but there are considerable differences between the USA and Europe in terms of the structure and funding of higher education, social and criminal justice systems, and the availability of treatment for substance use disorders. We therefore conducted a PRISMA-guided scoping review of the literature to answer the question ‘what is known from the existing literature about the use of illicit drugs by university students in the UK and Ireland?’. Scoping reviews aim to be comprehensive but with a focus on identifying gaps in the literature to inform policy. As such, they provide an overview of the research in an area of study but without an in-depth consideration of research quality [20]. The process involves identifying an initial research question, searching for and selecting relevant studies, and collating, charting, summarizing, and reporting the data [21]. This review is the first attempt to identify gaps in the evidence base to guide future research, policy and practice in identifying and reducing the potential harm of illicit psychoactive drug use in university students in the UK and Ireland.

## Methods

In line with scoping review guidance, we first considered the *concept* (what is known about illicit drug use), *target population* (university students in the UK or Ireland), and.*outcomes of interest* (including epidemiology, mechanisms of initiation, escalation and reduction in use, attitudes and beliefs about drugs, benefits or harms of use, and methods for detecting, preventing and treating problematic use) to clarify the focus of the scoping study [20, 22]. A search strategy was developed in line with our overarching question, and three electronic databases were searched in July 2021 to identify published papers: MEDLINE, PsycINFO and Web of Science. Search terms including ‘United Kingdom’, ‘Ireland’, ‘student’, ‘university’ and drug use’ were used (see Supplementary file 1 for a full list of search strings). The search terms were broad to be as comprehensive as possible. The reference lists of the included papers were searched and experts in the field contacted to identify any further evidence. The electronic databases EThOS and OpenGrey were used to search for unpublished evidence. Following the guidance for a systematic search created by the Canadian Agency for Drugs and Technology in Health [23], a general Google search was completed and the first 50 results were screened. There were no restrictions imposed on the date of publication, but due to time and cost restraints only English language papers were included.

### Study selection

The search produced 1583 potential papers for inclusion. The inclusion and exclusion criteria were formulated as an iterative process once the breadth of the literature was understood. Systematic reviews and literature reviews were excluded but reference lists were searched. Primary studies were not excluded based on their design, and both quantitative and qualitative research was included. Theses and student dissertations were included, but other unpublished literature was assessed separately. Studies that also included non-student participants or non-UK/Ireland-based universities were only included if the results were separated by population and the relevant data could be extracted. Papers discussing drug education in the university curriculum of healthcare professionals were excluded. The full search included papers that focussed on the use of drugs prescribed by a healthcare professional (even if used illicitly e.g. those used as ‘cognitive enhancers’). However, this report focuses on illicit drugs only (see data supplement 2 for excluded papers on the latter topic). If more than one report used duplicate data, the most comprehensive or relevant paper was included.

**Fig. 1:**
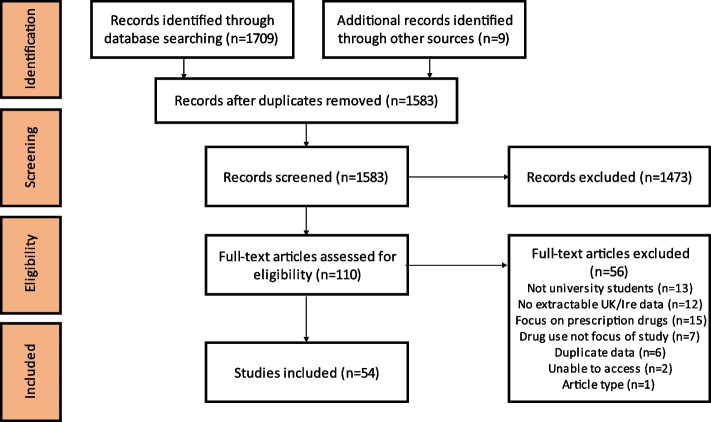
Preferred Reporting of Items for Systematic Reviews and Meta-Analyses (PRISMA) flowchart

Two independent reviewers (MB and ED) undertook the study selection process. The titles of the records found in the search were screened and the relevant abstracts independently assessed, with any disagreements between reviewers resolved through discussion. Full-text papers were then obtained and reviewed. The reviewers met frequently to discuss challenges surrounding study selection and to ensure the search strategy was suitable. Papers that assessed any aspect of the use of illicit drugs conducted in the UK or Ireland were included. Figure 1 shows the process of study selection using the Preferred Reporting Items for Systematic Reviews and Meta-Analyses (PRISMA) framework [24]. The excluded papers are listed in Supplementary file 2, along with the reasons for exclusion.

### Data extraction

An iterative model was used to determine the study characteristics extracted [20]. Both researchers independently used a data-charting tool to extract study characteristics from the first five papers included, before meeting to discuss any difficulties and refine the variables to extract. The final characteristics recorded were [1] author, [2] year of publication, [3] journal, [4] location of data collection, [5] study design, [6] delivery method (e.g., online survey, in-person, postal survey), [7] participant number, [8] response rate, [9] participant course of study, [10] participant year of study, [11] participant degree level (i.e., undergraduate, postgraduate), [12] participant gender, [13] participant age, [14] time-period assessed (e.g., lifetime, current use, past 12 months), [15] primary aim [16] primary outcome (categorized into 6 groups: prevalence and patterns of drug use, risk and protective factors, consequences of drug use, attitudes and knowledge, motivations for drug use, source of drugs), [17] ethical approval, and [18] funding source. One researcher (MB) then extracted the 18 study characteristics from each included study and the other researcher independently reviewed the completed data-charting form, with any disputes resolved with discussion. Quality was not formally assessed but ethical approval and funding were used as crude proxies in line with scoping review guidance [25].

A review of the grey literature found eleven relevant papers, all of which focussed on the prevalence of drug use among university students. One was a survey conducted by the UK National Union of Students and a national charity providing expertise on drugs and the law (Release) [11], and a second was commissioned by the UK Higher Education Policy Institute from a professional surveying organisation (YouthSight) [26]. A third reported on a national student survey in Ireland, and the remainder were student newspaper reports. The largest survey was carried out by The Tab, an online magazine covering youth culture and student issues. This had 16,000 responses from students across the UK, but no information was provided on the sampling methodology used. None of these papers reported how they recruited participants or their demographic characteristics, and so these studies are not considered further in this review but are detailed in Supplementary file 2.

## Results

Fifty-four peer-reviewed papers were included representing unique data from 50 separate studies, and 56 papers were excluded at the full-text review stage. These results are summarised in a PRISMA flowchart in Fig. 1.

### Study characteristics

Table 1 summarises the study characteristics. Since the 1980s the number of published papers has increased with each full decade, and almost half of the papers included in this review were published within the last 10 years. All four nations of the UK (England, Wales, Scotland, Northern Ireland) and the Republic of Ireland were represented. Five papers did not report their location within the UK, and only seven papers included universities in more than one country. English (44%) and Irish (19%) universities were the best represented. The majority (63%) of papers recruited participants from just one university, and only 3 (6%) recruited participants from 10 or more universities.

**Table 1:** Characteristics of the included studies

	Study variable	Number of studies
Year of Publication	1970 – 1979	7
	1980 – 1989	2
	1990 – 1999	9
	2000 – 2009	12
	2010—2019	23
	2020 onwards	1
Data collection methods	Quantitative -In-person questionnaire -Online questionnaire -Postal questionnaire -In-person and online questionnaires	47 31 9 6 1
	Qualitative interviews	4
	Mixed methods	3
Number of universities included*	1	34
	2–4	3
	5–9	7
	10 +	3
	Not reported	7
Location of university	England	24
	Wales	4
	Scotland	4
	Northern Ireland	0
	Republic of Ireland	10
	Great Britain	4
	UK (Great Britain & Northern Ireland)	3
	Not reported	5
Degree level	100% undergraduate	22
	Mixed undergraduate and graduate	8
	Nor reported	24
% of sample female	30–39	3
	40–49	10
	50–59	12
	60–69	11
	70–79	6
	80–89	2
	Not reported	10
% response rate	0–19	5
	20–39	4
	40–59	3
	60–79	11
	80–100	14
	Not applicable	4
	Not reported	13

The subject that participants were studying varied, with 16 (30%) papers limiting the sample to a particular course or courses (usually medical, dental or nursing studies). The academic year of study of participants was recorded in 41 (76%) of the papers, and the choice of year group was usually decided pragmatically with no particular focus on students early or late in their course of study. The gender breakdown in the 43 (80%) papers that reported it was between 30 and 89% female. The mean age of the sample was reported in 21 (39%) papers and ranged between 18.8 and 24.9 years.

### Study design

A clear majority of the studies had a cross-sectional design, with only 4 reporting data from more than one time point. Forty-seven papers (87%) reported a quantitative analysis, 4 (7%) reported a qualitative analysis and 3 (6%) used mixed methods. In those that employed mixed methods, cross-sectional data was used to inform later semi-structured interviews and open questionnaires. In the 51 papers where numerical data were collected, 36 (67%) used in-person interviews, 9 (17%) on-line data collection, 6 (11%) postal data collection and 1 analysed secondary data.

### Ethical approval and funding source

Under half (23, 43%) of the papers reported that they had obtained ethical approval and the remainder did not report whether approval had been sought or not. Twelve of the papers reported funding from various sources including university funds, the British Medical Association, the Wellcome Trust and the Northern Regional Health Authority. The remainder either did not report whether funding was received or specified that there was no external funding for the project.

### Drugs assessed

The drugs assessed in the papers were grouped into categories: a broad definition of illicit drugs (sometimes including prescription drugs used in an illicit way) (43, 80% of papers), cannabis only (8, 15%), and ecstasy only (3, 6%). Most papers included questions about drugs alongside alcohol and tobacco. Cannabis was the most reported drug under study, but the range of substances reported increased over time.

### Primary and secondary outcome domains

The outcomes of the papers were coded into five categories, with papers that reported more than one outcome coded multiple times. Forty-one of the fifty-four papers (76%) reported the prevalence or pattern of drug use, 28 (52%) factors associated with drug use, 14 (26%) student attitudes towards, knowledge about, and motivations to use drugs, 6 (11%) information about the source and supply of drugs, and 7 (13%) the consequences of drug use. The included papers are categorised by outcome in Table 2.

**Table 2:** Summary of the studies included in the review

Author	Location	Design	Method	*N*	% Undergraduate	Course	Response rate	% female	Mean age (range)	Substances examined	Prevalence	Knowledge/ Attitudes / Motivation	Associated factors	Consequences	Supply
**Hindmarch 1970 [**75**]**	1 uni England	Quantitative—Cross sectional	In-person	153	NR	NR	NR	NR	NR	Cannabis, heroin, LSD, amphetamines, other		X	X		
**McKay 1973 [**76**]**	1 uni Scotland	Quantitative—Cross sectional	In-person	749 (1971) 487 (1972)	78 73	Medical students	95.3 (1971) 80.6 (1972)	NR	NR	Marijuana, hashish, amphetamines, LSD, heroin, cocaine, barbiturates, glue, others	X		X		
**Einstein 1975 [**77**]**	1 uni England	Quantitative—Cross sectional	Postal survey	300	NR	NR	33	NR	NR	Cannabis	X		X		
**Hindmarch 1975 [**27**]**	1 uni England	Quantitative—Cross sectional	Postal survey	300	NR	NR	33	NR	NR	Cannabis		X			
**Parfrey 1975 [**33**]**	I uni Ireland	Quantitative—Cross sectional	Postal survey	444	100	Not limited	97	40	NR	Marijuana, LSD, ‘barbiturates or amphetamines’, ‘heroin or some hard drug’	X		X		X
**Somekh 1976 [**78**]**	6 unis England	Quantitative—Cross sectional	In-person	1113	100	Not limited	65	NR	20.9	Any drugs other than given by your doctor	X		X		
**Herity 1977 [**79**]**	1 uni Ireland	Quantitative—Cross sectional	In-person	765	NR	Medical students	98.6	33.3	NR	Pot, hashish, amphetamines, LSD, heroin, cocaine, barbiturates, glue, others	X		X		
**Golding 1987 [**45**]**	NR	Quantitative—Cross sectional	In-person	360	100 (Med students)NR (non-MS)	Medical and Non-medical (arts, science, agriculture)	84 (MS) 60 (non-MS)	NR	20.2	Cannabis and other drugs	X		X		
**Engs 1987 [**80**]**	4 colleges Scotland	Quantitative—Cross sectional	In-person	209	NR	Nursing students	100	89	NR	Marijuana, analgesics & other drugs	X		X		
**Ghodse 1994 [**81**]**	13 medical schools (Eng, Scot, Wales)	Quantitative—Cross sectional	Postal survey	1268	NR	Medical students	68	48	21.4 (17–35)	Cannabis, tranquillisers, cocaine, opiates, hallucinogens, barbiturates, amphetamines, ‘other drugs not taken under medical supervision’	X				
**Ashton 1995 [**43**]**	1 uni England	Quantitative—2 Cross-sectional studies (2 cohort samples collected a year apart)	In-person	186	100	Medical students	98.9	58.6	20.4	Cannabis, amphetamine, amyl nitrite, LSD, mescaline, ecstasy, magic mushrooms, cocaine, opioids, solvents/gas/glue	X		X		
**Webb 1996 [**49**]**	10 unis (Eng, Scot, Wales)	Quantitative—Cross sectional	In-person	3075	100	Specified—mixture	Almost 100%	47	20.9 (18–65)	Cannabis, LSD, amphetamines, cocaine/crack, ecstasy, magic mushrooms, amyl nitrate, barbiturates, heroin/morphine/opium, steroids, other drugs	X	X	X		
**Webb 1997 [**51**]**	10 unis (Eng, Scot)	Quantitative—Cross sectional	In-person	3699	100	Specified—mixture	Almost 100%	48.3	NR	Cannabis, LSD, amphetamines, cocaine/crack, ecstasy, magic mushrooms, amyl nitrate, barbiturates, heroin/morphine/opium, steroids, other drugs	X		X		
**Conner 1998 [**65**]**	England – unclear no. of unis	Quantitative—Cross sectional	In-person	186	NR	Not specified	62	58	NR (19–25)	Ecstasy	X	X	X		
**Webb 1998 [**50**]**	7 universities in GB	Quantitative—Cross sectional	In-person	785	100	Medical students	Almost 100%	55.3	20	Cannabis, LSD, amphetamines, cocaine/crack, ecstasy, magic mushrooms, amyl nitrate, barbiturates, heroin/morphine/opium, steroids, other drugs	X	X	X		
**Sell 1998 [**29**]**	1 uni England	Quantitative—Cross sectional	Postal survey	318	NR	NR	76	44	NR	Cannabis, LSD, Magic mushroom, Amphetamine, Ecstasy, Solvent/poppers, Cocaine, Sedatives, Crack cocaine, Heroin	X	X			X
**Makhoul 1998 [**28**]**	1 uni in England	Quantitative—cross-sectional	In-person	348	100	9 courses: psychology, law, computer studies, building surveying & housing studies, business studies, government, fashion, marketing, sociology	‘negligible'	64.1	23.3	Cannabis, amphetamines, hallucinogens (e.g. LSD), ecstasy, magic mushrooms, nitrites, cocaine, non-Px tranquilisers, heroin and opium derivatives, solvents, others	X	X			
**Engs 1999 [**82**]**	5 unis in Scotland	Quantitative: Cross sectional	In-person	4065	100	Not limited	92.2	76.7	21.6	Cannabis, amphetamines, cocaine, LSD, ecstasy, heroin	X		X		
**Newbury-Birch 2000 [**48**]**	1 uni in England	Quantitative: Cross sectional	In-person	194	100	Medical students	94.6	67	18.8 (17–39)	Cannabis, LSD, amphetamines, cocaine/crack, ecstasy, magic mushrooms, amyl nitrate, barbiturates, heroin/morphine/opium, steroids, other drugs	X		X		
**Pickard 2000 [**83**]**	1 uni in England	Quantitative: Cross sectional	In-person	136	100	Medical students	80.5	66.2	NR	Cannabis, amphetamines, LSD, ecstasy, amyl/butyl nitrate, magic mushrooms	X				
**Underwood 2000 [**84**]**	Not reported	Quantitative: cross-sectional	In-person	200	100	Dental students	75.8	NR	NR	Cannabis, amphetamines, amyl nitrate, ecstasy, magic mushrooms, LSD, cocaine, inhalants	X				
**Newbury-Birch 2001 [**47**]**	1 uni in England	Quantitative: longitudinal	In-person	122 (1995), 114 (1998)	100	Medical students	80.3% (1995) 79.7 (1998)	65.6% (1995) 66.7% (1998)	NR	Cannabis, LSD, amphetamines, cocaine/crack, ecstasy, magic mushrooms, amyl nitrate, barbiturates, heroin/morphine/opium, steroids, other drugs	X		X		
**McMillan 2002 [**30**]**	1 uni in England	Quantitative: longitudinal	In-person	380	100	Not limited	63	54.2	NR (19–53)	Amphetamine, cannabis, LSD, ecstasy	X	X			
**Newbury-Birch 2002 [**46**]**	1 uni in England	Quantitative: longitudinal	In-person	47 (2^nd^ yr 1995) 53 (final yr 1998)	100	Dental students	71.2% (1995) 80.3% (1998)	66% (1995) 49.1% (1998)	NR	Cannabis, LSD, amphetamines, cocaine/crack, ecstasy, magic mushrooms, amyl nitrate, barbiturates, heroin/morphine/opium, steroids, other drugs	X		X		
**Butler 2004 [**85**]**	1 uni in England	Quantitative: Cross sectional	In-person	254	100	Psychology	99.2	78.7%	22.3 (F) 21.5 (M)	Cannabis, amphetamine, cocaine, LSD, magic mushrooms, opiates, amyl nitrate, other	X		X		
**Terry 2005 [**41**]**	2 unis in England	Mixed methods: cross-sectional with results informing qualitative	In-person	176	NR	Science	N/A	43.4%	43.5%	Cannabis and ‘other illicit drugs’		X		X	
**Barber 2006[**86**]**	1 UK University	Quantitative—Cross sectional	In-person	346	NR	Dental (*n* = 218) Law (*n* = 128) students	78.1	54% dental, 59% law	21.4 dental, 20.4 law	Cannabis, ecstasy, amphetamines, ‘other class A drugs’, ‘other class B and class C drugs’	X		X		
**Boland 2006 [**87**]**	1 uni Ireland	Quantitative -longitudinal	In-person	1824	NR	Medical students	98.6 (1973) 95.6 (1990) 94.2 (2002)	33.3 (1973) 51.9 (1990) 59% (2002)	NR	‘Drugs for other than medical reasons’	X		X		
**Hammersley 2006 [**39**]**	> 1 uni England	Qualitative-structured interviews	In-person	176	NR	NR	N/A	43	22	Cannabis				X	
**Vivancos 2008 [**88**]**	1 uni England	Quantitative-prospective cohort	Online survey	827	NR	not limited	6	68	NR	Cannabis, opiates, amphetamines, crack, cocaine, benzodiazepines, solvents, other	X		X		
**Bartholomew 2010 [**38**]**	> 1 uni England	Quantitative—Cross sectional	In-person	143	100	Not specified	NR	58.9	18–24	Cannabis				X	
**Roberts 2010 [**89**]**	2 unis England	Quantitative—Cross sectional	In-person	360	90	Not limited	65	70	21.1 (M) 24.3 (F)	Magic mushrooms, ecstasy, LSD, amphetamines, cannabis, tranquilizers, cocaine, heroin, solvents, injected drugs (any type)			X		
**Underwood 2010 [**90**]**	1 English uni	Quantitative—Cross sectional	In-person	265	100	Dental students	69	Not reported	NR	Cannabis, amphetamines, amyl nitrate, ecstasy, magic mushrooms, LSD, cocaine, inhalants, steroids	X		X		
**Cahill 2010 [**91**]**	1 uni Ireland	Quantitative—Cross sectional	In-person	181	NR	Not specified	91.4	75.9	NR	Cannabis, cocaine, ecstasy, heroin, crystal meth, drugs by injection	X				
**Fisk 2011 [**42**]**	2 unis England	Quantitative—Cross sectional	Secondary data analysis	159	NR	NR	NR	49.7	21.6	Ecstasy, amphetamine, cannabis, cocaine				X	
**Skinner 2011 [**40**]**	1 uni Ireland	Quantitative—Cross sectional	In-person	1049	NR	Not limited	NR	82	21.2 (17–54)	Cannabis, ecstasy, cocaine, LSD, magic mushrooms, heroin	X			X	
**Houghton 2011 [**92**]**	1 uni Ireland	Quantitative—Cross sectional	In-person	760	NR	NR	76	48	22.2 (17–63)	Tranquillisers ± prescription, amphetamine, LSD, cocaine, heroin, ecstasy, drugs by injection, solvents, magic mushrooms, cannabis	X				
**Deasy 2014 [**93**]**	1 uni Ireland	Mixed methods—cross-sectional with results informing semi-structured interviews	In-person	1030 (survey) 59 (interview)	NR	Teacher education and nursing/midwifery	71	54.6	NR	Cannabis	X	X	X		
**Pedersen 2014 [**31**]**	1 uni UK	Quantitative—Cross sectional	In-person	473	100	Social Sciences	NR	NR	NR	Cannabis	X	X			
**Bennett 2014 [**37**]**	South Wales	Quantitative—Cross sectional	Online survey	2181	100	Not limited	33	59	NR	Cannabis, ecstasy, LSD, magic mushrooms, amphetamine, cocaine, ketamine, heroin, crack, methamphetamine, mephedrone, GBL/GHB, Spice, BZP	X			X	
**El Ansari 2015 [**15**]**	7 unis England, Wales, NI	Quantitative—Cross sectional	In-person	3706	NR	Random selection	80	77.9	24.9	‘Illicit substance’ (includes ecstasy, marijuana, cocaine, heroin, crack, LSD, amphetamines)	X		X		
**Deniozou 2015 [**94**]**	1 uni Scotland	Quantitative—Cross sectional	Online survey	1189	84	Not limited	15	68	NR	Ecstasy, cannabis, cocaine, Ketamine, magic mushroom, LSD, benzodiazepines, amyl nitrate, heroin, amphetamines, crack, methamphetamine, GHB, steroids, 2CB, mephedrone, prescribed medication, NPS, other	X				
**Coomber 2016 [**35**]**	1 uni NR	Qualitative—semi-structured interviews	N/A	30	100	including but not limited to: sociology, business studies and geology	N/A	NR	NR	Cocaine, ecstasy, cannabis, Ketamine, diazepam, mushrooms, mephedrone					X
**Holt 2016 [**95**]**	1 uni NR	Quantitative—Cross sectional	Online survey	3683	NR	not limited	11.5	68.1	NR	Not fully reported but includes cannabis, ecstasy, cocaine, amyl nitrate, nitrous oxide, DMT, solvents, injecting drug use	X				
**Zvauyu 2017 [**96**]**	1 uni England	Quantitative—Cross sectional	Paper – drop off	410	41.4	Graduate entry (GE) and undergraduate medical students	49.1	GE 56.3 UG 68.8	GE: 22–26 UG: 18–21	‘Drug use’		X			
**Bennett 2018 [**36**]**	7 unis in Wales	Quantitative—Cross sectional	Online survey	7855	NR	Not limited	7.8	62.3	NR	Cannabis, ecstasy, LSD, magic mushrooms, amphetamine, methamphetamine, cocaine powder, crack cocaine, heroin, and tranquilizers				X	
**Patton 2018 [**16**]**	1 uni in England	Mixed methods—cross sectional with quotes from participants	In-person and online	512	91	Social sciences (sociology, psychology, criminology, politics, international relations)	41.2	62	NR	Amphetamine, amyl nitrate, cannabis, cocaine, ecstasy, ketamine, LSD, heroin, methadone, crack, methamphetamine, modafinil, Adderall, Ritalin, dexedrine, ephedrine, BZP, khat, mephedrone, Spice, GBL/GHB, other new psychoactive Substances	X	X			X
**Bogowicz 2018 [**44**]**	1 uni in England	Quantitative—Cross sectional	In-person	1242	NR	Medical and law students	78.8	51.6	NR	Amphetamines, anabolic steroids, benzodiazepines or Z-drugs, cannabis, cathinones, cocaine, ecstasy, GBL/GHB, ketamine, LSD, mushrooms, nitrous oxide, opioids, piperazines, synthetic cannabinoids	X		X		
**Holloway 2018 [**97**]**	7 unis in Wales	Quantitative—Cross sectional	Online survey	7855	NR	All students	7.8	62.3	NR	Cannabis, ecstasy, LSD, magic mushrooms, amphetamine, methamphetamine, cocaine powder, crack cocaine, heroin, and tranquilizers	X		X		
**Bickerdike 2019 [**98**]**	1 uni in Ireland	Quantitative—Cross sectional	Online survey	2267	90.2	Not limited	20.1	51.7	NR	Amphetamine, cocaine, ecstasy/MDMA, ‘head shop products’, heroin, LSD, magic mushrooms, solvents, tranquilisers, drugs by injection	X				
**Bennett 2019 [**34**]**	7 unis in Wales	Quantitative—Cross sectional	Online survey	1877	NR	NR	NR	NR	NR	Cannabis, ecstasy, LSD, magic mushrooms, amphetamine, methamphetamine, cocaine powder, crack cocaine, heroin, and tranquilizers					X
**Moyle 2019 [**9**]**	1 uni in England	Qualitative—semi-structured interviews	NA	30	100	Not limited	NA	30	NR (18–37)	Cocaine, ecstasy, cannabis, Ketamine, diazepam, mushrooms, mephedrone					X
**Murphy 2019 [**99**]**	Various unis in Ireland	Quantitative—Cross sectional	Online survey	5672	95.1	Not limited	NR	48.7	21.6	‘Non-prescribed/recreational’	X		X		
**Lane 2020 [**100**]**	1 uni in Ireland	Qualitative—free-text survey	NA	161	59.6	Medical students	NA	54.6	24.8 (22–42)	‘Drugs’—not specified		X			

*NR* Not reported

### Prevalence or patterns of drug use

The 41 papers examining prevalence or patterns of illicit drug use collected data through either an in-person interview (29, 71%), a postal response (4, 10%), an online survey (7, 17%) or both in-person and online methods (1, 2%). The number of participants ranged from 47 to 7855, and 39 of the 46 (85%) samples reported included a percentage response rate ranging from 6 to 100%. The response rate varied by the method used to collect data (in-person 60–100%, postal 33–97%, online 6–33%, in-person and online 41%). In terms of participants recruited the online surveys had the most participants (mean of 3382 compared with mean of 765 in the in-person surveys and 430 in the postal surveys) but the lowest response rates (mean of 15.5% compared with 82.2% in-person and 68.5% postal). A range of time periods of drug use were assessed, including lifetime (35 papers), past year [13], past 6 months [2], past 3 months [1], past 3 months [1], past 30 days [1], past month [1], past 4 weeks [1], past week [3], ‘since starting degree’ [1], ‘current academic year’ [1] and ‘current’ [12]. There was a broad trend towards students reporting more experience of a wider range of illicit substances. However, the variability in participant samples, the methods used to collect the data, and the time periods of drug use considered meant that it was not possible to formally assess trends in drug use over time.

### Factors associated with drug use

Twenty-eight papers (52%) assessed factors associated with drug use, and these are summarised in Table 3. Many papers reported more than one associated factor. Twelve papers (22%) explored demographic variables, including age, gender, ethnicity, socioeconomic status, living circumstances and international student status. Eleven (20%) measured personality factors or mental health, including instruments measuring sensation seeking and anxiety. The link between health-related behaviours, including tobacco smoking, alcohol consumption and physical activity, and drug use was assessed in 11 (20%) papers. Other associations included academic course or year of study (8, 15%), attitudes to drug use and health awareness (3, 6%), normative beliefs (2, 4%), academic performance (2, 4%) and religious beliefs (2, 4%). No clear and consistent patterns of association could be drawn from the data.

**Table 3:** Factors associated with drug use

**Author**	**Year**	**Location**	**Study design**	N	Number of unis	Demographics	Course/ Year of study	Personality or mental health	Attitudes/ health awareness	Normative beliefs	Tobacco or alcohol use	Academic performance	Religious beliefs
Ashton [43]	1995	England	Cross-sectional	186	1			**X**					
Barber [86]	2006	Not reported	Cross-sectional	346	1		**X**						
Boland [87]	2006	Ireland	Longitudinal	1824	1	**X**							
Bogowicz [44]	2018	England	Cross-sectional	1242	1	**X**	**X**	**X**			**X**		
Butler [85]	2004	England	Cross-sectional	254	1			**X**					
Conner [65]	1998	England	Cross-sectional	186	Not reported				**X**	**X**			
Deasy [93]	2014	Ireland	Mixed methods: cross-sectional & semi-structured interviews	1030 (survey)	1			**X**					
Einstein [77]	1975	Not reported	Cross-sectional	300	1						**X**		
El Ansari [15]	2015	England, Wales and Northern Ireland	Cross-sectional	3706	7	**X**		**X**	**X**		**X**	**X**	**X**
Engs [80]	1987	Scotland	Cross-sectional	209	3		**X**						
Engs [82]	1999	Scotland	Cross-sectional	4065	Not reported						**X**		**X**
Golding [45]	1987	Not reported	Cross-sectional	360	1		**X**	**X**					
Herity [79]	1977	Ireland	Cross-sectional	765	1	**X**					**X**		
Hindmarch [75]	1970	Not reported	Cross-sectional	153	1	**X**			**X**			**X**	
Holloway [97]	2018	Wales	Cross-sectional	7855	7	**X**	**X**						
McKay [76]	1973	Scotland	Cross-sectional	1236	1	**X**					**X**		
Murphy [99]	2019	Ireland	Cross-sectional	5672	31	**X**	**X**						
Newbury-Birch [48]	2000	England	Cross-sectional	194	1			**X**			**X**		
Newbury-Birch [47]	2001	England	Longitudinal	236	1	**X**		**X**			**X**		
Newbury-Birch [46]	2002	England	Longitudinal	100	1			**X**			**X**		
Parfrey [33]	1975	Ireland	Cross-sectional	444	1					**X**			
Roberts [89]	2010	England	Cross-sectional	360	2	**X**							
Somekh [78]	1976	England	Cross-sectional	1113	6	**X**	**X**						
Underwood [90]	2010	England	Cross-sectional	265	1						**X**		
Vivancos [101]	2009	England	Cross-sectional	827	1	**X**							
Webb [49]	1996	England, Scotland, Wales	Cross-sectional	3075	10			**X**					
Webb [51]	1997	England, Scotland	Cross-sectional	3699	10		**X**						
Webb [50]	1998	Not reported	Cross-sectional	785	7			**X**			**X**		

### Attitudes towards, knowledge about, and motivations to use illicit drugs

Fourteen papers assessed student attitudes towards drugs and/or their knowledge about their effects. Issues covered included the morality or ethics of drug use, safety beliefs, the perceived effect of drug use and perceived motivations for use. Early studies noted that attitudes to cannabis were markedly different to those towards tobacco or alcohol [27]. People that drank alcohol perceived that people that used cannabis were ‘definitely emotionally unstable’ and ‘definitely less able to cope with life’, whereas the latter group perceived that people that used illicit drugs in general were ‘more interested’ and ‘vested with more friends’. This attitudinal separation was hypothesised to be an effective ‘barrier’ to starting cannabis use.

By the late 1990s, papers were reporting that students that regularly used illicit drugs were similar to the general population of students both in their views about the causes of drug use and their personal and social characteristics [28]. Both students that used drugs and those that did not agreed that youth culture influences and sensation-seeking were the most endorsed reasons for drug taking. The illegality of drugs had little influence on levels of consumption [29]. Some support was found to support the idea that increasingly liberal views towards drugs would appear across a student’s time at university [30]. Although attitudes towards tobacco became less positive in year 3 when compared to starting university, there was no such change for illicit drugs. By the 2010s, students were shown to rate tobacco as most harmful to physical health, alcohol most harmful with regard to injuries and social consequences, and cannabis as most harmful with regard to mental health [31]. As the legal substance alcohol was rated as more harmful than the illegal substance cannabis, the authors hypothesised that young people in the years to come may be less supportive of a traditional drug policy based on criminalization [31].

By 2018, Patton’s survey found that the top three reasons for drug consumption were for fun or pleasure, for relaxation, and to enhance an activity [16]. These reasons were thought to fit with the ‘normalization’ hypothesis [32]. Depictions of drug use in the media were widespread, and 78% felt comfortable consuming media that featured drug use. There was also further evidence of the shift from drug use as a deviant activity into mainstream cultural arrangements (59% of abstainers had one or more close friends who use drugs) [16]. These results suggest that attitudes towards drugs may have changed over time amongst the student population, but the level of acceptance is not uniform or consistent between different substances in different populations.

### The supply of illicit drugs

Six papers were concerned with the supply of drugs. An early study in the 1970s in Ireland found that most students were approached to buy drugs at parties, in pubs or hotels, or at clubs. Roughly half of students obtained their first drugs from friends [33]. A study in England in the 1990s also found that drugs were usually bought from friends and were most commonly consumed in other people’s rooms or at parties [29]. The authors contrasted this with alcohol which was consumed in bars or public places. More recently, a larger survey of 7 of the 9 universities in Wales found that half of the students that used drugs obtained them solely from friends and associates, and another 25% used friends and external markets [34]. In many cases supplying drugs amounted to sharing them or giving them away, but over a third said they had sold drugs. Drugs like nitrous oxide, cannabis, synthetic cannabinoids, ecstasy and magic mushrooms were usually sourced from friends, whereas other drugs (khat, crack, steroids, heroin) more likely to be bought. Male students were more likely to buy from dealers. The authors concluded that methods used by university students to obtain and supply drugs shared features of both ‘social supply’ and ‘traditional drug markets’ [34].

Moyle and Coomber also considered the nature of the supply of drugs in students. They conducted semi-structured interviews with 60 social suppliers of recreational drugs in two studies involving both a student population (*n* = 30) and a general population sample (*n* = 30) [9, 35]. Both samples provided evidence that supplying drugs to, and receiving them from, friends and social contacts had become increasingly normalised and seen as less than ‘real dealing’ and more like gift-giving [35]. Early experiences of social supply occurred pre-university and usually involved a ‘one off’ act of sharing cannabis. However, once at university this had increased to ‘buying cannabis in bulk and selling excess amounts to friends, and/or purchasing ‘standard’ 3.5 g bags of powders like cocaine and MDMA on behalf of a group and retaining a quantity of the substance as payment’. This behaviour rarely continued when they returned home [9].

### Consequences of drug use

Seven papers reported the consequences of drug use. Two studies surveyed consequences of any illicit drug [36, 37], four focussed on cannabis [38–41] and one on ecstasy [42]. Two large online surveys collected data on physical and psychological effects of drugs and drug-related crime, providing a broad overview of a range of issues. In contrast studies utilising in-depth structured interviews with qualitative analysis explored the positive and negative effects of cannabis [39] and the effects of cannabis on driving [41]. One lab-based study examined cannabis-related impairments in prospective memory by comparing people that used cannabis and people that did not on both self-reported prospective memory failures and on an objective video-based prospective memory task [38]. Two studies used objective measures to quantify the consequences of drug use on mental health e.g., the Hospital Anxiety and Depression scale [40, 42]. Overall, the positive effects of relaxation, mood elevation and enhanced creativity were balanced by negative effects such as forgetfulness, poor concentration, and reduced productivity. Impaired mental health was a common theme, including paranoia, moodiness, anxiety, irritability, confusion and dependence. Crime-related consequences included driving under the influence, antisocial behaviour and selling drugs [36, 41].

## Discussion

This scoping review was conducted at a time when increasing attention was being paid to the issue of drug use in university students, and media reports about drug-related student deaths had prompted a government response in both the UK and Ireland. Universities UK, a body representing 140 Universities across the UK, formed a ‘Task Force’ to explore the issue of drugs on campus in early 2022 [17], with the stated aim of setting out ‘*a common approach to reduce harms from drug use and to better tackle supply’*. The aim of this scoping review was to map the breadth and depth of research into illicit drug use in university students in the UK and Ireland. In this paper our focus was on illicit drugs, and we excluded a growing body of work on the illicit use of prescribed stimulant medications as aids to studying (to be analysed elsewhere). We made no attempt to assess the quality of studies included, and do not claim to draw conclusions about the findings. However, the gaps in the resulting survey of research covering a period of over 50 years may help to guide policy makers and researchers in the UUK Task Force.

### The epidemiology of drug use in university student populations

Monitoring student drug (and alcohol) use over time should be critical to the development of effective evidence-based policy and intervention strategies. High quality estimates can be used to identify trends and patterns, understand the direct and indirect harms of drug use, and guide further research to understand risk and protective factors for student drug use and the effectiveness of policy or treatment interventions. This review found that the prevalence of drug use was the most studied area in terms of number of published papers. However, as has been noted by other researchers [16], the existing UK/Irish research is methodologically limited. Most published studies reported prevalence of use, but the time window of assessment and the instruments used were variable. Studies including students from more than one university were the exception, and the population under study was often drawn from a single university department. It was rare for a paper to distinguish between single use (e.g., tried once in a lifetime) and regular use (e.g., several days in the past week), and validated clinical diagnoses were never reported. Most of the published papers described cross-sectional studies, with no attempt to follow up participants. One research group based in the north-east of England has repeated studies over time [43–51], but not used the same methodology or followed through one cohort for significant length of time. Little of the research moved beyond simple descriptions and correlations, and it was rare that any under-pinning theory or conceptual model was described. Finally, there was very little evidence about drug use in specific high-risk populations e.g., LGBTQ or non-white students, or students with co-existing mental health problems.

The Advisory Council on the Misuse of Drugs (ACMD) has noted that prevalence data on young people’s drug use within the UK are ‘generally limited, highly variable and of low quality’ [52]. They recommend reviewing the scope and detail of the current approaches to monitor prevalence, ensuring that outcome measures used are fit for purpose. Expertise and experience in this area can be drawn from North America, where regular national surveys using sound methodology include university-age students [12]. The authors of a Canadian report on quality standards for measuring drug use in high schools [53] noted that surveying students in school is an efficient and cost-effective means of collecting data from young people, and similar reasoning could be applied to universities. However, there is a need for standardised methodology that can be replicated over time in representative samples.

*Recommendation*: A national survey of student drug use that covers the whole of the UK and Northern Ireland would be helpful, particularly if it was repeated to monitor trends over time. Standardised data collection instruments tailored to young adults must be developed and tested (e.g. see [53]), and the first step towards this approach has already been taken in Ireland under government direction [54]. Alternatively, the creation of representative student ‘consumer panels’ may allow researchers to explore patterns and frequencies of drug use in nationally representative samples.

### Positive and negative risk factors for drug use

Although several studies described the association of a range of demographic, psychological and social factors with drug use, methodological limitations limited the utility of these findings. Some important areas of potential study were completely absent in our findings. For example, a developing evidence base suggests that there is a positive association between adverse childhood experiences (ACEs) and the development of substance use disorder in adolescence and adulthood [55]. One potential framework for study is the life course model of substance use [56], with a focus on the role of illicit drug use in developmental role transitions. Many authors have argued that emerging adulthood (i.e. the traditional university years) is developmentally different from adolescence (school) or full adulthood [10]. Independence from parents, new social and romantic relationships with peers, increased access to drugs and alcohol, and the need for self-directed study all contribute to a unique social milieu at a developmental stage already characterised by peak levels of risk-taking and high levels of mental health problems [57–59]. Understanding the factors that increase or decrease substance use at university is important to develop effective responses.

*Recommendation:* Prospective cohort study designs are needed that include student populations to identify which factors play a role in the initiation, development and cessation of drug use. Risk and protective factors for illicit drug use in young adults may be conceptualised as contextual (e.g. availability of the drug, or social norms that are tolerant of illicit drug use), fixed markers of risk (e.g., sex, parental and sibling substance use, poverty or potential genetic factors), and individual and interpersonal risk factors (e.g., novelty and sensation seeking, conduct disorder in childhood, parenting styles, or poor quality of parent–child interaction) [2, 60]. Affiliation with peers that use drugs is one of the strongest predictors of illicit drug use in young adults and of crucial importance in the transitional social milieu of a university campus [61]. The impact of an increasing awareness of neurodiversity in young adults should also be investigated.

### The harms (and benefits) of drug use in the student population

The consequences of illicit drug use were only reported in seven of the papers included in this review, with some limited focus on mental health and criminal justice issues. There were no studies exploring the impact on university-specific outcomes such as completion of a course of study, academic achievement and progression to further study or employment. This is surprising, as these outcomes are important markers of university quality used in national league tables. Understanding the specific harms that relate to university students will help to tailor prevention and treatment responses to this population.

*Recommendation*: The potential harms of illicit drug use in student populations occur across several domains, including academic performance (attendance and grades), other high-risk behaviours (unprotected sex, violence, driving under the influence), exacerbation of mental health problems, or legal issues (prosecution for possession or dealing) [62]. These consequences could potentially reshape the entire trajectory of the student’s life course [63]. Therefore, it would be useful to collect, collate and track standardised data nationally from a range of university departments (e.g., student welfare, registry) as a marker of illicit drug-related harm. Such quantitative data could usefully be supplemented by detailed qualitative studies of each aspect of harm (e.g., academic, physical, mental, social, or legal).

### Knowledge about, attitudes and motivation to use drugs

Levels of objective knowledge about drug use were rarely studied, and yet may form the bedrock of a harm reduction approach [64]. Likewise attitudes and motivations to use drugs were not often reported or studied, despite the existence of potentially useful underpinning theories such as the theory of planned behaviour [65]. The use of conceptual models to guide findings is important to build an effective evidence base for prevention and intervention. The 2021 UK Government Drug Strategy aims to achieve a ‘generational shift in the demand for drugs’, and one proposed strategy for achieving this is ‘*research and testing messaging through an evidence-based, targeted behaviour change initiative, initially aimed at students in further and higher education’* [66] (p49). The document further notes that communications campaigns work best when they are tailored and targeted to the audience, and a recent ACMD report on prevention of illicit drug use notes that some activities have been ineffective, such as fear arousal approaches (including ‘scared straight’ approaches) or stand-alone mass media campaigns [67].

Our results suggest that a national trend towards ‘normalisation’ of recreational drug use has been replicated on university campuses. Furthermore, this has merged with the social supply of drugs, leading to a perception amongst some students that supplying (sometimes) large amounts of drugs is routine [35]. However, the overall picture is complicated, and not all studies included students who didn’t use drugs and so their voice was often not heard. Survey work using student panels rather than open online questionnaires shows that some students believe that their university should take a tougher stance on drugs [26]. The ‘social norms’ approach is based on challenging misperceptions individuals hold about their peers. Research at eight Further Education Colleges in the UK reported a perceived norm of frequency of substance use that was higher than the reported norm, and the majority of respondents did not actively approve of tobacco, cannabis or other drug use [68]. This reflects similar findings in the university system in Canada [69]. The social norms approach may be a viable method of developing effective methods of behaviour change in UK students.

*Recommendation*: Studies of knowledge about, and attitudes towards, illicit drugs in representative populations of UK or Irish students would be helpful in designing strategies to educate students about illicit drug use. Theoretically-driven interventions to reduce use and prevent harm may have a significant impact later in life, and comparisons could usefully be drawn with non-student peers.

### Prevention and treatment of drug-related harms

Our scoping review found little evidence of published work in UK/Irish universities on prevention of, or intervention to reduce, drug-related harms. Reviews of the North American literature on prevention and treatment have also noted a lack of published studies, but parent-based and in-person brief motivational interventions appear to be promising [18, 70]. Our review found no such interventions published in UK or Irish student populations. This may reflect the slow response of universities to consider drug use and tackle its potential harms, and the impact of stigma and illegality on students’ help-seeking attempts. A review of psychological interventions for prevention and treatment of mental health disorders in university students [71] was also limited by the poor quality of the literature and exclusion of non-published data. It noted considerable uncertainty about the best way to provide interventions for students, and relatively few trials adapted intervention delivery to student-specific concerns. It called for further work to better understand the mechanisms underlying students’ mental health problems, perhaps using transdiagnostic, stepped care approaches. Research on both mental health and drug use should involve students in the design of interventions to increase their acceptability to this population.

The prevention of alcohol-related harm has been well studied in university populations, and several existing interventions for student drinking share theoretical and methodological underpinnings with effective interventions in drug prevention and treatment in other populations (i.e., school-based prevention, adolescent and adult drug treatment) [6, 18]. These interventions could be adapted to target drug prevention on university campuses. As is the case in the wider community, a recovery orientated system of care is required, with a full continuum of care encompassing harm reduction through to abstinence [72]. The development of the first Collegiate Recovery Programs [21] to support abstinent students at Teesside University and the University of Birmingham [73] represents the first part of such a continuum. However, despite big strides in the development of campus-based mental health support in the past decade, the issue of addiction to drugs, alcohol or other behaviours has been largely ignored. Tackling this deficit will be important to ensure that students are able to maximise the potential benefits of a university education.

*Recommendation*: Several commentators have noted that the stigma of psychoactive drug use appears to be particularly prominent in universities in the UK and Ireland, with ‘zero tolerance’ approaches often limiting informed debate [64]. Working collaboratively across the Higher Education sector may be helpful in supporting universities to provide education and prevent harm whilst respecting the illegality of illicit drug use. There is also a need to develop interventions tailored to the unique needs of students who have developed a drug use disorder, and to evaluate abstinence-based recovery programs on campus.

## Conclusions

This review has exposed large gaps in the research evidence base around illicit drug use in university students in the UK and Ireland. The limited evidence reviewed here suggests that more students are coming into contact with illicit drugs and many are experiencing harms. There is therefore a need to unite student unions and universities in exploring the prevalence of drug use and its impact on students, supported by high quality research. A national survey of student drug use that covers the whole of the UK and Northern Ireland would be helpful, particularly if it was repeated to monitor trends over time. Alternatively, the creation of representative student ‘consumer panels’ may allow researchers to understand attitudes of students to the use of psychoactive substances on campus and to explore methods of reducing harm. Little effort has been made to explore the views of those who do not use drugs, or to identify the motivations of university students to decrease or cease drug use. Promising areas of future research on motivations to change in relation to illicit drug use include the social contextual factors, perceptions of effects on social relationships, and actions of friends and family members to prompt contemplation of change [74]. Trials to evaluate novel theoretically-based prevention and treatment programs that take into account established risk factors for drug use and drug use disorders are also needed [18, 70].

## Supplementary Information

**Additional file 1. **

**Additional file 2: Table1. **Papers excluded from the study and reason for exclusion (*n*=40). **Table2. **Additional papers excluded because the focus was prescribed drugs used as‘cognitive enhancers’ and not illicit drugs (*n*=15). **Table3. **Grey Literature – all excluded as no detail about methods used.

## Data Availability

The datasets used and/or analysed during the current study are available from the corresponding author on reasonable request.

## Abbreviations

MDMA – 3,4: Methylenedioxymethamphetamine
HEI: Higher Education Institute
PRISMA: Preferred Reporting of Items for Systematic Reviews and Meta-Analyses

## References

[1] Babor TF, Caulkins J, Fischer B, Foxcroft D, Humphreys K, Medina-Mora ME (2018). Drug Policy and the Public Good.

[2] Degenhardt L, Stockings E, Patton G, Hall WD, Lynskey M (2016). The increasing global health priority of substance use in young people. Lancet Psychiatry.

[3] Pedrelli P, Nyer M, Yeung A, Zulauf C, Wilens T (2015). College Students: Mental Health Problems and Treatment Considerations. Acad Psychiatry.

[4] Office for National Statistics. Drug misuse in England and Wales: year ending March 2020 2020 [Available from: https://www.ons.gov.uk/peoplepopulationandcommunity/crimeandjustice/articles/drugmisuseinenglandandwales/yearendingmarch2020.

[5] Hall WD, Patton G, Stockings E, Weier M, Lynskey M, Morley KI, et al. Why young people’s substance use matters for global health. Lancet Psychiatry. 2016;3:265–79. 10.1016/S2215-0366(16)00013-4 26905482

[6] Stockings E, Hall WD, Lynskey M, Morley KI, Reavley N, Strang J (2016). Prevention, early intervention, harm reduction, and treatment of substance use in young people. Lancet Psychiatry.

[7] Arnett JJ (2005). The Developmental Context of Substance use in Emerging Adulthood. J Drug Issues.

[8] Rhodes T (2002). The ‘risk environment’: a framework for understanding and reducing drug-related harm. Int J Drug Policy.

[9] Moyle L, Coomber R. Student transitions into drug supply: exploring the university as a ‘risk environment.’ J Youth Stud. 2019;22(5):642–57.

[10] Schwartz SJ, Petrova M (2019). Prevention Science in Emerging Adulthood: a Field Coming of Age. Prev Sci.

[11] National Union of Students, Release. Taking the Hit: Student drug use and how Institutions respond. London: NUS; 2018. p. 7. Available from: https://www.release.org.uk/publications/taking-hit-student-drug-use-and-how-institutions-respond.

[12] Schulenberg JE, Johnston LD, O’Malley PM, Bachman JG, Miech RA, Patrick ME. Monitoring the Future national survey results on drug use, 1975–2019: Volume II, College students and adults ages 19–60. Ann Arbor: Institute for Social Research: The University of Michigan; 2020. p. 7.

[13] HM Government (2017). 2017 Drug Strategy.

[14] Government of Ireland. Framework for Response to the Use of Illicit Substances within Higher Education. Department of Education: Government of Ireland; 2020. p. 3.

[15] El Ansari W, Vallentin-Holbech L, Stock C (2015). Predictors of Illicit Drug/s Use Among University Students in Northern Ireland, Wales and England. Global J Health Sci.

[16] Patton D (2018). Navigating drugs at university: normalisation, differentiation and drift?. Safer Communities.

[17] Universities UK. Student drug use: reducing harm and tackling supply: UUK; 2022 [Available from: https://www.universitiesuk.ac.uk/latest/news/student-drug-use-reducing-harm-and.

[18] Larimer ME, Kilmer JR, Lee CM (2005). College Student Drug Prevention: A Review of Individually-Oriented Prevention Strategies. J Drug Issues.

[19] Skidmore CR, Kaufman EA, Crowell SE (2016). Substance Use Among College Students. Child Adolesc Psychiatr Clin N Am.

[20] Arksey H, O’Malley L. Scoping studies: towards a methodological framework. Int J Soc Res Methodol. 2005;8(1):19–32.

[21] Vest N, Reinstra M, Timko C, Kelly J, Humphreys K (2021). College programming for students in addiction recovery: A PRISMA-guided scoping review. Addict Behav.

[22] Levac D, Colquhoun H, O’Brien KK. Scoping studies: advancing the methodology. Implement Sci. 2010;5(1):69. 10.1186/1748-5908-5-69 PMC2954944 20854677

[23] The Canadian Agency for Drugs and Technologies in Health. Grey matters: a practical tool for searching health-related grey literature [Internet]. Ottawa: CADTH; 2018. p. 3. Available from: https://www.cadth.ca/grey-matters-practical-tool-searching-health-related-grey-literature.

[24] Page MJ, McKenzie JE, Bossuyt PM, Boutron I, Hoffmann TC, Mulrow CD (2021). The PRISMA 2020 statement: an updated guideline for reporting systematic reviews. BMJ.

[25] Daudt HML, van Mossel C, Scott SJ (2013). Enhancing the scoping study methodology: a large, inter-professional team’s experience with Arksey and O’Malley’s framework. BMC Med Res Methodol.

[26] Hillman N. Most students think taking illegal drugs causes problems for users as well as society and want their universities to take a tougher stance: Higher Education Policy Institute; 2018. Available from: https://www.hepi.ac.uk/2018/06/12/students-think-taking-illegal-drugs-causes-problems-users-well-society-want-universities-take-tougher-stance/.

[27] Hindmarch I, Hughes I, Einstein R. Attitudes to drug users and to the use of alcohol, tobaccoand cannabis on the campus of a provincial university. UNODC Bulletin on Narcotics. 1975;1:27–36. 1039281

[28] Makhoul M, Yates F, Wolfson S (1998). A survey of substance use at a UK university: prevalence of use and views of students. J Addict Med.

[29] Sell L, Robson P (1998). Perceptions of College Life, Emotional Well-being and Patterns of Drug and Alcohol Use among Oxford Undergraduates. Oxf Rev Educ.

[30] McMillan B, Conner M (2002). Drug Use and Cognitions About Drug Use Amongst Students: Changes Over the University Career. J Youth Adolesc.

[31] Pedersen W, Grip Fjær E, Gray P, von Soest T (2014). Perceptions of Harms Associated With Tobacco, Alcohol, and Cannabis Among Students From the UK and Norway. Contemp Drug Probl.

[32] Parker H (2005). Normalization as a barometer: Recreational drug use and the consumption of leisure by younger Britons. Addict Res Theory.

[33] Parfrey PS (1975). Intoxicant Use among University Students in Cork. J Ir Med Assoc.

[34] Bennett T, Holloway K (2019). How Do Students Source and Supply Drugs? Characteristics of the University Illegal Drug Trade. Subst Use Misuse.

[35] Coomber R, Moyle L, South N (2016). The normalisation of drug supply: The social supply of drugs as the “other side” of the history of normalisation.. Drugs Educ Prevention Policy.

[36] Bennett T, Holloway K (2018). Drug and Alcohol-Related Crime Among University Students. Int J Offender Ther Comp Criminol.

[37] Bennett TH, Holloway KR (2014). Drug misuse among university students in the UK: implications for prevention. Subst Use Misuse.

[38] Bartholomew J, Holroyd S, Heffernan TM (2010). Does cannabis use affect prospective memory in young adults?. J Psychopharmacol.

[39] Hammersley R, Leon V (2006). Patterns of cannabis use and positive and negative experiences of use amongst university students. Addict Res Theory.

[40] Skinner R, Conlon L, Gibbons D, McDonald C (2011). Cannabis use and nonclinical dimensions of psychosis in university students presenting to primary care. Acta Psychiatr Scand.

[41] Terry P, Wright KA (2005). Self-reported driving behaviour and attitudes towards driving under the influence of cannabis among three different user groups in England. Addict Behav.

[42] Fisk JE, Murphy PN, Montgomery C, Hadjiefthyvoulou F. Modelling the adverse effects associated with ecstasy use Addiction 2011;106:798-805. 10.1111/j.1360-0443.2010.03272.x 21182557

[43] Ashton CH, Kamali F (1995). Personality, lifestyles, alcohol and drug consumption in a sample of British medical students. Med Educ.

[44] Bogowicz P, Ferguson J, Gilvarry E, Kamali F, Kaner E, Newbury-Birch D (2018). Alcohol and other substance use among medical and law students at a UK university: a cross-sectional questionnaire survey. Postgrad Med J.

[45] Golding JF, Cornish AM (1987). Personality and life-style in medical students: Psychopharmacological aspects. Psychol Health.

[46] Newbury-Birch D, Lowry RJ, Kamal F (2002). The changing patterns of drinking, illicit drug use, stress, anxiety and depression in dental students in a UK dental school: a longitudinal study. Br Dent J.

[47] Newbury-Birch D, Walshaw D, Kamali F (2001). Drink and drugs: from medical students to doctors. Drug Alcohol Depend.

[48] Newbury-Birch D, White M, Kamali F (2000). Factors influencing alcohol and illicit drug use amongst medical students. Drug Alcohol Depend.

[49] Webb E, Ashton CH, Kelly P, Kamali F (1996). Alcohol and drug use in UK university students. The Lancet.

[50] Webb E, Ashton CH, Kelly P, Kamali F. An update on British medical students’ lifestyles. Med Educ. 1998;32:325–31. 10.1046/j.1365-2923.1998.00204.x 9743790

[51] Webb E, Ashton H, Kelly P, Kamali F (1997). Patterns of alcohol consumption, smoking and illicit drug use in British university students: interfaculty comparisons. Drug Alcohol Depend.

[52] Bowden-Jones O, Finch E, Campbell A. Re: ACMD Vulnerable Groups - Young People’s Drug Use. London: Advisory Council on the Misuse of Drugs; 2022.

[53] Student Drug Use Surveys Working Group. The Value of student alcohol and drug use surveys. Ottawa, Canada: Canadian Centre on Substance Abuse; 2013. p. 3. Available from: https://ccsa.ca/sites/default/files/2019-04/SDUS-Value-en.pdf.

[54] Byrne M, Dick S, Ryan L, Dockray S, Davoren M, Heavin C (2022). The Drug Use in Higher Education in Ireland (DUHEI) Survey 2021: Main Findings.

[55] Leza L, Siria S, López-Goñi JJ, Fernández-Montalvo J (2021). Adverse childhood experiences (ACEs) and substance use disorder (SUD): A scoping review. Drug Alcohol Depend.

[56] Hser Y-I, Longshore D, Anglin MD (2007). The life course perspective on drug use: A conceptual framework for understanding drug use trajectories. Eval Rev.

[57] Cadigan JM, Duckworth JC, Parker ME, Lee CM (2019). Influence of developmental social role transitions on young adult substance use. Curr Opin Psychol.

[58] Hammond CJ, Mayes LC, Potenza MN (2014). Neurobiology of adolescent substance use and addictive behaviors: treatment implications. Adolesc Med State Art Rev.

[59] Ibrahim AK, Kelly SJ, Adams CE, Glazebrook C (2013). A systematic review of studies of depression prevalence in university students. J Psychiatr Res.

[60] Stone AL, Becker LG, Huber AM, Catalano RF (2012). Review of risk and protective factors of substance use and problem use in emerging adulthood. Addict Behav.

[61] Fergusson DM, Boden JM, Horwood LJ (2008). The developmental antecedents of illicit drug use: evidence from a 25-year longitudinal study. Drug Alcohol Depend.

[62] Vasiliou VS, Dockray S, Dick S, Davoren MP, Heavin C, Linehan C (2021). Reducing drug-use harms among higher education students: MyUSE contextual-behaviour change digital intervention development using the Behaviour Change Wheel. Harm Reduct J.

[63] Schuster C, O’Malley PM, Bachman JG, Johnston LD, Schulenberg J. Adolescent marijuana use and adult occupational attainment: A longitudinal study from age 18 to 28. Subst Use Misuse. 2001;36(8):997–1014. 10.1081/ja-100104486 11504156

[64] Ozcubukcu A, Towl G (2022). Illicit drug use in universities: zero tolerance or harm reduction?.

[65] Conner M, Sherlock K, Orbel S (1998). Psychosocial determinants of ecstasy use in young people in the UK. Br J Health Psychol.

[66] HM Government. From Harm to Hope: A 10-year drugs plan to cut crime and save lives. London; 2021. p. 49. Available from: https://assets.publishing.service.gov.uk/government/uploads/system/uploads/attachment_data/file/1079147/From_harm_to_hope_PDF.pdf. 10.1016/j.drugpo.2022.103840 36068144

[67] Advisory Council on the Misuse of Drugs (2022). Drug Misuse Prevention Review.

[68] McAlaney J, Jenkins W (2017). Perceived social norms of health behaviours and college engagement in British students. J Furth High Educ.

[69] Loverock A, Yakovenko I, Wild TC (2021). Cannabis norm perceptions among Canadian university students. Addict Behav.

[70] Dennhardt AA, Murphy JG (2013). Prevention and treatment of college student drug use: A review of the literature. Addict Behav.

[71] Barnett P, Arundell L-L, Saunders R, Matthews H, Pilling S (2021). The efficacy of psychological interventions for the prevention and treatment of mental health disorders in university students: A systematic review and meta-analysis. J Affect Disord.

[72] Ashford RD, Brown AM, Ryding R, Curtis B (2020). Building recovery ready communities: the recovery ready ecosystem model and community framework. Addict Res Theory.

[73] Trainor L (2023). Being better than well at the University of Birmingham. J Subst Use Addict Treat.

[74] Whelan E, Dockray S, Dick S, Davoren MP, Heavin C, Linehan C (2020). Motivations to decrease and cease substance use in third-level students: A scoping review.

[75] Hindmarch I (1970). Patterns of Drug Use in a Provincial University. Br J Addict.

[76] McKay AJ, Hawthorne VM, McCartney HN (1973). Drug Taking Among Medical Students at Glasgow University. BMJ.

[77] Einstein R, Hughes IE, Hindmarch I (1975). Patterns of Use of Alcohol, Cannabis and Tobacco in a Student Population. Br J Addict.

[78] Somekh D (1976). Prevalence of Self-Reported Drug Use among London Undergraduates. Br J Addict.

[79] Herity B, Wilson-Davis K, Horgan JM, Bourke GJ (1977). Tobacco, Alcohol and other Drug use among Medical Students. J Ir Med Assoc.

[80] Engs RC, Rendell KH (1987). Alcohol, tobacco, caffeine and other drug use among nursing students in the Tayside Region of Scotland: a comparison between first- and final-year students. Health Educ Res: Theory Pract.

[81] Ghodse AH, Howse K (1994). Substance use of medical students: a nationwide survey. Health Trends.

[82] Engs RC, Mullen K (1999). The Effect of Religion and Religiosity on Drug Use Among a Selected Sample of Post Secondary Students in Scotland. Addict Res.

[83] Pickard M, Bates L, Dorian M, Greig H, Saint D (2000). Alcohol and drug use in second-year medical students at the University of Leeds. Med Educ.

[84] Underwood B, Fox K (2000). A survey of alcohol and drug use among UK based dental undergraduates. Br Dent J.

[85] Butler GKL, Montgomery AMJ (2004). Impulsivity, risk taking and recreational ‘ecstasy’ (MDMA) use. Drug Alcohol Depend.

[86] Barber MW, Fairclough A (2006). A comparison of alcohol and drug use among dental undergraduates and a group of non-medical, professional undergraduates. Br Dent J.

[87] Boland M, Fitzpatrick P, Scallan E, Daly L, Herity B, Horgan J (2006). Trends in medical student use of tobacco, alcohol and drugs in an Irish university, 1973–2002. Drug Alcohol Depend.

[88] Vivancos R, Abubakar I, Hunter PR (2008). Sex, drugs and sexually transmitted infections in British university students. Int J STD AIDS.

[89] Roberts R, Golding J, Towell T, Weinreb I (2010). The Effects of Economic Circumstances on British Students’ Mental and Physical Health. J Am Coll Health.

[90] Underwood B, Fox K, Manogue M (2010). Tobacco, alcohol and drug use among dental undergraduates at one English university in 1998 and 2008. Br Dent J.

[91] Cahill E, Byrne M (2010). Alcohol and Drug Use in Students Attending a Student Health Centre. Ir Med J.

[92] Houghton F, Keane N, Murphy S, Houghton C, Dunne C (2011). 12 Month Prevalence of Drug Use Among Third-Level Students in Limerick City. Ir Med J.

[93] Deasy C, Coughlan B, Pironom J, Jourdan D, Mannix-McNamara P (2014). Psychological distress and lifestyle of students: implications for health promotion. Health Promot Int.

[94] Deniozou T (2015). Student health and lifestyle survey.

[95] Holt M, Powell S (2016). Healthy Universities: a guiding framework for universities to examine the distinctive health needs of its own student population. Perspect Public Health.

[96] Zvauya R, Oyebode F, Day EJ, Thomas CP, Jones LA (2017). A comparison of stress levels, coping styles and psychological morbidity between graduate-entry and traditional undergraduate medical students during the first 2 years at a UK medical school. BMC Res Notes.

[97] Holloway K, Bennett T (2018). Characteristics and correlates of drug use and misuse among university students in Wales: a survey of seven universities. Addict Res Theory.

[98] Bickerdike A, Dinneen J, O’Neill C (2019). ‘A Healthy CIT’: An Investigation into Student Health Metrics, Lifestyle Behaviours and the Predictors of Positive Mental Health in an Irish Higher Education Setting. Int J Environ Res Public Health.

[99] Murphy JJ, MacDonncha C, Murphy MH, Murphy N, Timperio A, Leech RM (2019). Identification of health-related behavioural clusters and their association with demographic characteristics in Irish university students. BMC Public Health.

[100] Lane A, McGrath J, Cleary E, Guerandel A, Malone KM (2020). Worried, weary and worn out: mixed-method study of stress and well-being in final-year medical students. BMJ Open.

[101] Vivancos R, Abubakar I, Hunter PR (2009). Sexual behaviour, drugs and alcohol use of international students at a British university: a cross-sectional survey. Int J STD AIDS.

