# Supplementary material for: Illicit drug use in university students in the UK and Ireland: a PRISMA-guided scoping review
Source: Subst Abuse Treat Prev Policy. 2023 Mar 14;18:18. doi: 10.1186/s13011-023-00526-1 (PMC10012457; doi:10.1186/s13011-023-00526-1)
Supplement: Supplementary file 1 — Additional file 1. [file 13011_2023_526_MOESM1_ESM.docx]

**Supplementary File 1**

**Literature Search Strategies**

All searches run on 24/07/2021

Medline:

mp = (United Kingdom OR UK OR England OR Wales OR Ireland OR Northern Ireland OR Scotland OR Britain) AND ti,kw = (Student* OR Undergraduate* OR Universit* OR College) AND mp = (Drug* OR Substance-Related Disorders OR Prescription Drug* OR Cognitive enhancer OR Nootropic Agents OR Substance* OR Illicit drug OR Cannabis OR Marijuana OR Cocaine OR Inhalants OR MDMA OR Amphetamine OR Opioid OR Hallucinogens OR Ecstasy)

PsycINFO:

mp = (United Kingdom OR UK OR England OR Wales OR Ireland OR Northern Ireland OR Scotland OR Britain) AND mp = (Student* OR College OR College Students OR Undergraduate) AND mp = (exp Drugs OR exp Prescription Drugs OR exp Nootropic Drugs OR exp Performance Enhancing Drugs OR exp Nonprescription Drugs OR exp Substance Abuse and Addiction Measures OR exp Substance Use Disorder OR exp Substance Related and Addictive Disorders OR Drug abuse OR exp Cannabis OR exp Cannabis Use Disorder OR Marijuana OR exp Cocaine OR exp Crack Cocaine OR exp Drug Dependency OR exp Drug Addiction OR exp Inhalant Abuse OR exp Opiates OR exp Lysergic Acid Diethylamide OR exp Hallucinogenic Drugs OR exp methylenedioxymethamphetamine)

Web Of Science:

TS = (United Kingdom OR UK OR England OR Wales OR Ireland OR Northern Ireland OR Scotland OR Britain) AND TS = (Student* OR Undergraduate* OR Universit* OR College) AND TI,AK,KP = (Drug* OR Substance-Related Disorders OR Prescription Drug* OR Cognitive enhancer OR Nootropic Agents OR Substance* OR Illicit drug OR Cannabis OR Marijuana OR Cocaine OR Inhalants OR MDMA OR Amphetamine OR Opioid OR Hallucinogens OR Ecstasy)

OpenGrey:

(United Kingdom OR UK OR England OR Wales OR Ireland OR Northern Ireland OR Scotland OR Britain) AND (Student* OR Undergraduate* OR Universit* OR College) AND keyword:(Drug* OR Substance-Related Disorders OR Prescription Drug* OR Cognitive enhancer OR Nootropic Agents OR Substance* OR Illicit drug OR Cannabis OR Marijuana OR Cocaine OR Inhalants OR MDMA OR Amphetamine OR Opioid OR Hallucinogens or Ecstasy)

EThOS:

‘student drug UK’ and ‘student drug Ireland’
