# Supplementary material for: Illicit drug use in university students in the UK and Ireland: a PRISMA-guided scoping review
Source: Subst Abuse Treat Prev Policy. 2023 Mar 14;18:18. doi: 10.1186/s13011-023-00526-1 (PMC10012457; doi:10.1186/s13011-023-00526-1)
Supplement: Supplementary file 2 — Additional file 2: Table1. Papers excluded from the study and reason for exclusion (n=40). Table2. Additional papers excluded because the focus was prescribed drugs used as‘cognitive enhancers’ and not illicit drugs (n=15). Table3. Grey Literature – all excluded as no detail about methods used. [file 13011_2023_526_MOESM2_ESM.docx]

**Supplementary File 2**

**PRISMA diagram for the full search**


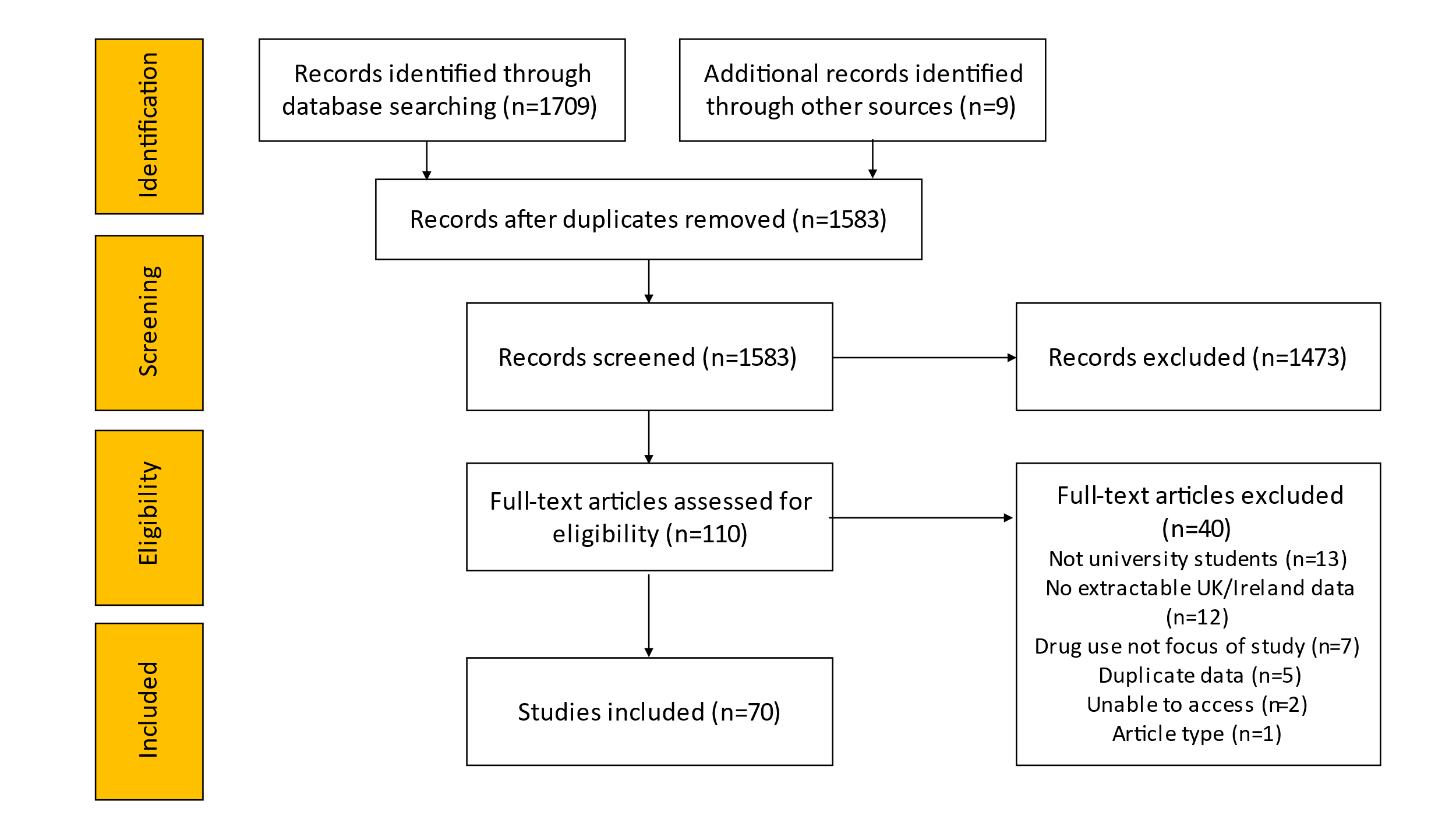


**Table 1. Papers excluded from the study and reason for exclusion (n=40)**

| **Reference** | **Reason for exclusion** |
| --- | --- |
| 1. Anonymous. Students’ attitudes to drugs. | Unable to access full-text |
| 1. Ansong MA, Moody ML, Stachnik J. Fee-for-service drug information centers. Drug information journal: DIJ/Drug Information Association. 2003 Apr;37(2):233-9. | Not assessing university students |
| 1. Auerbach RP, Mortier P, Bruffaerts R, Alonso J, Benjet C, Cuijpers P, Demyttenaere K, Ebert DD, Green JG, Hasking P, Lee S. Mental disorder comorbidity and suicidal thoughts and behaviors in the world health organization world mental health surveys international college student initiative. International journal of methods in psychiatric research. 2019 Jun;28(2):e1752. | Not UK or Ireland: Also includes non-UK students and doesn’t report separately |
| 1. Auerbach RP, Mortier P, Bruffaerts R, Alonso J, Benjet C, Cuijpers P, Demyttenaere K, Ebert DD, Green JG, Hasking P, Murray E. WHO world mental health surveys international college student project: prevalence and distribution of mental disorders. Journal of abnormal psychology. 2018 Oct;127(7):623. | Not UK or Ireland: Also includes non-UK students and doesn’t report separately |
| 1. Banerjee SC, Greene K, Yanovitzky I. Sensation seeking and dosage effect: An exploration of the role of surprise in anti-cocaine messages. Journal of Substance Use. 2011 Feb 1;16(1):1-3. | Focus not drug use: University students were convenience sample. |
| 1. Banerjee SC, Greene K. ‘I quit’ versus ‘I’m sorry I used’: A preliminary investigation of variations in narrative ending and transportation. Psychology & health. 2012 Nov 1;27(11):1308-22. | Focus not drug use: University students were convenience sample. |
| 1. Bennett TH. Differences in the age‐drug use curve among students and non‐students in the UK. Drug and Alcohol Review. 2014 May;33(3):280-6. | Not assessing university students: Also includes non-students and doesn’t report separately |
| 1. Bennett TH, Holloway KR. Drug use among college and university students: Findings from a national survey. Journal of Substance Use. 2015 Jan 2;20(1):50-5. | Not assessing university students: Also includes non-students and doesn’t report separately |
| 1. Corazza O, Simonato P, Corkery J, Trincas G, Schifano F. “Legal highs”: safe and legal “heavens”? A study on the diffusion, knowledge and risk awareness of novel psychoactive drugs among students in the UK. Rivista di psichiatria. 2014 Mar 1;49(2). | Not assessing university students: Also includes non-students and doesn’t report separately |
| 1. Dargan PI, Albert S, Wood DM. Mephedrone use and associated adverse effects in school and college/university students before the UK legislation change. QJM: An International Journal of Medicine. 2010 Nov 1;103(11):875-9. | Not assessing university students: Also includes non-students and doesn’t report separately |
| 1. Deligianni E, Daniel OJ, Corkery JM, Schifano F, Lione LA. Impact of the UK Psychoactive Substances Act on awareness, use, experiences and knowledge of potential associated health risks of novel psychoactive substances. British journal of clinical pharmacology. 2020 Mar;86(3):505-16. | Not assessing university students: Also includes non-students and doesn’t report separately |
| 1. Dempsey RC, McAlaney J, Helmer SM, Pischke CR, Akvardar Y, Bewick BM, Fawkner HJ, Guillen-Grima F, Stock C, Vriesacker B, Van Hal G. Normative perceptions of cannabis use among European university students: associations of perceived peer use and peer attitudes with personal use and attitudes. Journal of studies on alcohol and drugs. 2016 Sep;77(5):740-8. | Not UK or Ireland: Also includes non-UK students and doesn’t report separately |
| 1. Dunne C, Somerset M. Health promotion in university: what do students want?. Health Education. 2004 Dec 1. | Focus is not drug use |
| 1. El Ansari W, Oskrochi R, Haghgoo G. Are students’ symptoms and health complaints associated with perceived stress at university? Perspectives from the United Kingdom and Egypt. International journal of environmental research and public health. 2014 Oct;11(10):9981-10002. | Duplicate data |
| 1. El Ansari W, Stock C, John J, Deeny P, Phillips C, Snelgrove S, Adetunji H, Hu X, Parke S, Stoate M, Mabhala A. Health promoting behaviours and lifestyle characteristics of students at seven universities in the UK. Central European journal of public health. 2011 Dec 1;19(4):197-204. | Duplicate data |
| 1. Engs RC, Van Teijlingen E. Correlates of alcohol, tobacco and marijuana use among Scottish postsecondary helping-profession students. Journal of studies on alcohol. 1997 Jul;58(4):435-44. | Duplicate data |
| 1. Erickson K, Backhouse SH, Carless D. “I don't know if I would report them”: Student-athletes' thoughts, feelings and anticipated behaviours on blowing the whistle on doping in sport. Psychology of Sport and Exercise. 2017 May 1;30:45-54. | Not UK or Ireland: Also includes non-UK students and doesn’t report separately |
| 1. French DP, James DH. Reasons for the use of mild analgesics among English students. Pharmacy World & Science. 2008 Jan;30(1):79-85. | Focus not drug use: Not use of psychoactive drugs |
| 1. Gunn AD. Drug abuse and the student. The Practitioner. 1974 Jun;212(1272):830-3. | Unable to access full-text |
| 1. Hansen WB, Fleming CB, Scheier LM. Self-reported engagement in a drug prevention program: Individual and classroom effects on proximal and behavioral outcomes. The journal of primary prevention. 2019 Feb;40(1):5-34. | Not assessing university students |
| 1. Helmer SM, Pischke CR, Van Hal G, Vriesacker B, Dempsey RC, Akvardar Y, Guillen-Grima F, Salonna F, Stock C, Zeeb H. Personal and perceived peer use and attitudes towards the use of nonmedical prescription stimulants to improve academic performance among university students in seven European countries. Drug and alcohol dependence. 2016 Nov 1;168:128-34. | Not UK or Ireland: Also includes non-UK students and doesn’t report separately |
| 1. Johnson JL, Evers KE, Paiva AL, Van Marter DF, Prochaska JO, Prochaska JM, Mauriello LM, Cummins CO, Padula JA. Prevention profiles: understanding youth who do not use substances. Addictive Behaviors. 2006 Sep 1;31(9):1593-606. | Not assessing university students |
| 1. Kosviner A, Hawks D. Cannabis Use amongst British University Students: II. Patterns of Use and Attitudes to Use. British Journal of Addiction to Alcohol & Other Drugs. 1977 Mar;72(1):41-57. | Not assessing university students: Also includes non-students and doesn’t report separately |
| 1. Kosviner A, Hawks D, Webb MG. Cannabis use amongst British university students: Prevalence rates and differences between students who have tried cannabis and those who have never tried it. British Journal of Addiction. 1974 Mar. | Not assessing university students: Also includes non-students and doesn’t report separately |
| 1. Lamb A. UK dental undergraduates–a survey of alcohol and drug use. British Dental Journal. 2000 Sep;189(6):307-. | Article type: Editorial |
| 1. Main DJ, Boon JC, McAllister H. Law and Psychology in the Perception of Crime Seriousness: Towards a Joint Approach. Psychology, Law, and Criminal Justice 2011 Jun 15 (pp. 401-406). De Gruyter. | Not assessing university students: Also includes non-students and doesn’t report separately |
| 1. Martens MP, Page JC, Mowry ES, Damann KM, Taylor KK, Cimini MD. Differences between actual and perceived student norms: An examination of alcohol use, drug use, and sexual behavior. Journal of American college health. 2006 Mar 1;54(5):295-300. | Not UK or Ireland |
| 1. Martin CS, Clifford PR, Clapper RL. Patterns and predictors of simultaneous and concurrent use of alcohol, tobacco, marijuana, and hallucinogens in first-year college students. Journal of Substance Abuse. 1992 Jan 1;4(3):319-26. | Not UK or Ireland |
| 1. McAlaney J, Dempsey RC, Helmer SM, Van Hal G, Bewick BM, Akvardar Y, Guillén-Grima F, Orosová OG, Kalina O, Stock C, Zeeb H. Negative Consequences of substance use in European university students: Results from Project SNIPE. European addiction research. 2021;27(1):75-82. | Not UK or Ireland: Also includes non-UK students and doesn’t report separately |
| 1. Menahem S. Cardiac asystole following cannabis (marijuana) usage–additional mechanism for sudden death?. Forensic science international. 2013 Dec 10;233(1-3):e3-5. | Not UK or Ireland |
| 1. Moreno M, Estevez AF, Zaldivar F, Montes JM, Gutiérrez-Ferre VE, Esteban L, Sánchez-Santed F, Flores P. Impulsivity differences in recreational cannabis users and binge drinkers in a university population. Drug and alcohol dependence. 2012 Aug 1;124(3):355-62. | Not UK or Ireland |
| 1. Mosher CE, Danoff-Burg S. Indoor tanning, mental health, and substance use among college students: the significance of gender. Journal of health psychology. 2010 Sep;15(6):819-27. | Not UK or Ireland |
| 1. O’Connor S, McCaffrey N, Whyte E, Moran K, Lacey P. Nonsteroidal anti-inflammatory drug use, knowledge, and behaviors around their use and misuse in Irish collegiate student-athletes. The Physician and Sportsmedicine. 2019 Jul 3;47(3):318-22. | Focus not drug use: Non-psychoactive medication |
| 1. Parfrey PS. Factors associated with undergraduate marijuana use in Cork. British Journal of Addiction to Alcohol & Other Drugs. 1977 Mar;72(1):59-65. | Duplicate data |
| 1. Parrott AC, Sisk E, Turner JJ. Psychobiological problems in heavy ‘ecstasy’(MDMA) polydrug users. Drug and Alcohol Dependence. 2000 Jul 1;60(1):105-10. | Not assessing university students |
| 1. Pezaro S, Patterson J, Moncrieff G, Ghai I. A systematic integrative review of the literature on midwives and student midwives engaged in problematic substance use. Midwifery. 2020 Oct 1;89:102785. | Not assessing university students: Also includes non-students and doesn’t report separately |
| 1. Raab GM, Burns SM, Scott G, Cudmore S, Ross A, Gore SM, OʼBrien F, Shaw T. HIV prevalence and risk factors in university students. AIDS. 1995 Feb. | Focus not drug use |
| 1. Richardson T, Gallagher A, Garavan H. Cannabis use and psychotic experiences in an international sample of undergraduate students. Psychosis. 2011 Jun 1;3(2):141-4. | Not UK or Ireland: Also includes non-UK students and doesn’t report separately |
| 1. Vivancos R, Abubakar I, Hunter PR. Sex, drugs and sexually transmitted infections in British university students. International journal of STD & AIDS. 2008 Jun;19(6):370-7. | Duplicate data |
| 1. Zhao J, Chen Y, Han T, Westland S. Designing effective warnings about addiction on the patient information leaflet of over-the-counter codeine sold in England to university students. International Journal of Environmental Research and Public Health. 2020 Jan;17(15):5490. | Focus not drug use: University students used as a convenience sample |

**Table 2. Additional papers excluded because the focus was prescribed drugs used as ‘cognitive enhancers’ and not illicit drugs (n=15)**

| **Reference** |
| --- |
| 1. Adamopoulos P, Ho H, Sykes G, Szekely P, Dommett EJ. Learning Approaches and Attitudes Toward Cognitive Enhancers in UK University Students. Journal of Psychoactive Drugs. 2020;52(3):248-54. |
| 1. Bennett TH, Holloway KR, Brookman F, Parry O, Gorden C. Explaining prescription drug misuse among students from a widening access university: The role of techniques of neutralization. Drugs: Education, Prevention and Policy. 2014;21(3):189-96 |
| 1. Champagne J, Gardner B, Dommett EJ. Modelling predictors of UK undergraduates’ attitudes towards smart drugs. Trends in Neuroscience and Education. 2019;14:33-9 |
| 1. Coveney CM. Awakening Expectations: exploring social and ethical issues surrounding the medical and non-medical uses of cognition enhancing drugs in the UK: The University of Nottingham; 2010 |
| 1. Goulding E, Murphy M, Di Blasi Z. Sharing and borrowing prescription medication: a survey of Irish college students. Ir J Med Sci. 2011;180:687-90 |
| 1. Holloway K, Bennett T. Prescription drug misuse among university staff and students: A survey of motives, nature and extent. Drugs: Education, Prevention and Policy. 2012;19(2):137-44 |
| 1. Holloway KR, Bennett TH, Parry O, Gorden C. Characteristics and consequences of prescription drug misuse among university students in the United Kingdom. Journal of Substance Use. 2014;19(1-2):156-63 |
| 1. McDermott H, Lane H, Alonso M. Working smart: the use of ‘cognitive enhancers’ by UK university students. Journal of Further and Higher Education. 2021;45(2):270-83 |
| 1. Nguyen NT, Rakow T, Gardner B, Dommett EJ. Understanding the relationship between safety beliefs and knowledge for cognitive enhancers in UK university students. PLoS ONE. 2021;16(1):e0244865 |
| 1. Singh I, Bard I, Jackson J. Robust Resilience and Substantial Interest: A Survey of Pharmacological Cognitive Enhancement among University Students in the UK and Ireland. . PLoS ONE. 2014;9(10):e105969 |
| 1. Steward A, Pickersgill M. Developing expertise, customising sleep, enhancing study practices: exploring the legitimisation of modafinil use within the accounts of UK undergraduate students. Drugs: Education, Prevention and Policy. 2019;26(4):347-55 |
| 1. Tully JL. Exploring the Aetiology and Effects of Pharmacological Cognitive Enhancement use in UK University Students. Liverpool: Liverpool John Moores University; 2020 |
| 1. Vagwala MK, Bicquelet A, Didziokaite G, Coomber R, Corrigan O, Singh I. Towards a Moral Ecology of Pharmacological Cognitive Enhancement in British Universities. Neuroethics. 2017;10:389-402 |
| 1. Vargo EJ, James RA, Agyeman K, MacPhee T, McIntyre R, Ronca F, et al. Perceptions of assisted cognitive and sport performance enhancement among university students in England. Performance Enhancement & Health. 2014;3(2):66-77 |
| 1. Vargo EJ, Petróczi A. “It Was Me on a Good Day”: Exploring the Smart Drug Use Phenomenon in England. . Frontiers in Psychology. 2016;7:779 |

**Table 3. Grey Literature – all excluded as no detail about methods used**

| **Author** | **Title** | **Year** | **Article Type** | **Number of students surveyed** | **Source** |
| --- | --- | --- | --- | --- | --- |
| [NUS](https://www.release.org.uk/sites/default/files/pdf/publications/Taking%20the%20Hit%20-%20Student%20drug%20use%20and%20how%20institutions%20respond%20-.pdf) and Release | Taking the Hit Student drug use and how Institutions respond | 2018 | Student society research | 2810 | Reference search |
| [The Tab](https://thetab.com/uk/2020/12/22/16000-students-took-our-drugs-survey-find-out-how-and-what-theyre-taking-this-term-187354) | The Tab’s 2020 Drugs Survey | 2020 | Newspaper study | 16,000 | Google search |
| [YouthSight](https://www.hepi.ac.uk/2018/06/12/students-think-taking-illegal-drugs-causes-problems-users-well-society-want-universities-take-tougher-stance/) with University of Buckingham | Survey of attitudes towards drugs among full-time undergraduate students | 2018 | Higher Education Policy Institute | 1059 | Google search |
| [Tim Bingham](https://www.drugsandalcohol.ie/24807/1/National%20Student%20Drug%20Survey%202015.pdf), Colin O'Driscoll & Graham De Barra | National Student Drug Survey | 2015 | Report | 2701 | Reference search |
| [Jo Barrow](https://www.yorkvision.co.uk/archived/eat-sleep-work-repeat/14/01/2014) | Eat sleep work repeat: are York students succumbing to study drugs like modafinil? | 2014 | Newspaper study | 240 | Reference search |
| [Daniel Kodsi](https://cherwell.org/2016/05/13/revealed-oxfords-addiction-to-study-drugs/#:~:text=According%20to%20a%20Cherwell%20survey,prescription%20while%20studying%20at%20Oxford.) | Revealed: Oxford’s addiction to study drugs | 2016 | Newspaper study | 662 | Reference search |
| [Natasha Lennard](http://archive.varsity.co.uk/693.pdf) | One in ten takes drugs to study | 2009 | Newspaper study | 1000 | Reference search |
| [Max Long](https://cherwell.org/2014/01/24/investigation-drugs-in-oxford/) | Investigation: drugs in Oxford. | 2014 | Newspaper study | 650 | Reference search |
| [Louis Mercier](https://www.oxfordstudent.com/2015/06/04/almost-one-in-five-oxford-students-use-study-drugs-survey-reveals/#:~:text=A%20survey%20conducted%20by%20The,work%20or%20prepare%20for%20exams.&text=However%2C%20the%20lack%20of%20research,university%20policy%20from%20taking%20fruition.) & Cason Reily | Almost one in five Oxford students use study drugs, survey reveals. | 2015 | Newspaper study | 207 | Reference search |
| [Josephine Rabinowitz](https://www.cherwell.org/2012/05/30/smart-drugs-on-offer-to-students/) | ‘Smart drugs’ on offer to students | 2012 | Newspaper study | Not reported | Reference search |
| [Student Beans](https://www.theguardian.com/education/interactive/2012/oct/12/university-drug-culture-survey) | Drug culture at UK universities: study findings | 2012 | Newspaper study | 1093 | Reference search |
